# The Diagnostic Utility of Ultrasound in Myxofibrosarcoma: Insights From a Multimodal Imaging Case Study

**DOI:** 10.1055/a-2826-3276

**Published:** 2026-04-16

**Authors:** Neil Limaye, Hannah Masraf, Henry Conchie, Adrian Lim

**Affiliations:** 1 8946 Radiology, Imperial College Healthcare NHS Trust, London, United Kingdom of Great Britain and Northern Ireland; 2 4970 Radiology, Royal Marsden Hospital NHS Trust, London, United Kingdom of Great Britain and Northern Ireland

## Introduction

Myxofibrosarcoma (MFS) is a malignant soft tissue sarcoma, representing less than 1% of all adult malignancies (Xiao et al. in *Frontiers in Oncology* . 2024). MFS often arises in extremities, especially the lower limbs, and is less common in the torso or head and neck. Clinically, this can present as a painless mass with slow rates of growth but often demonstrates a high propensity for infiltration and recurrence. MFS is characterised by pleomorphic spindle cells with a myxoid stroma and often demonstrates variable histological architectures and unique imaging signs (Vanni et al. *The Adv Med Oncol* . 2022). We describe a case that demonstrates the benefits of multi-modality imaging when investigating complex soft tissue lesions, underscoring the utility of ultrasound in characterising malignant sarcomas, enabling prompt referral and management.

## Case description

A 65-year-old patient with a history of radiotherapy-treated cervical cancer presented to the emergency department with a 2-week history of a rapidly growing, painful left groin lump. Physical examination revealed a fixed, palpable 7–8 cm soft tissue lump in the left medial inguinal region. The laboratory results demonstrated an increased C-reactive protein level of 58.3 mg/L. The white cell count (7.4×10^9/L) and the remaining blood parameters were within normal ranges. The clinical team’s initial working differential diagnosis was either an abscess, haematoma or infected/metastatic lymphadenopathy from prior cervical cancer.

**Fig. 1:**
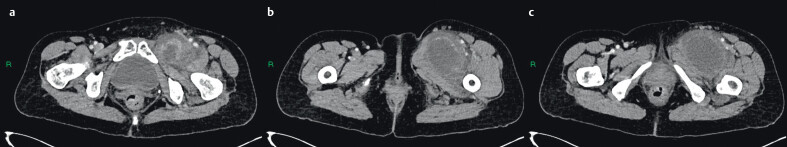
**(a–c)** Contrast-enhanced portal-venous phase axial CT of the abdomen and pelvis imaging demonstrates a heterogenous, peripherally enhancing, mixed density lesion with adjacent inflammatory fat stranding and lateral displacement of the femoral vessels. CT, computed tomography.

The patient underwent contrast-enhanced computed tomography (CT) of the abdomen and pelvis, which demonstrated a heterogenous, peripherally enhancing, hypoattenuating lesion with a small soft tissue component superiorly, initially favouring an abscess in the context of raised inflammatory markers ( Fig. 1 ).

**Fig. 2:**
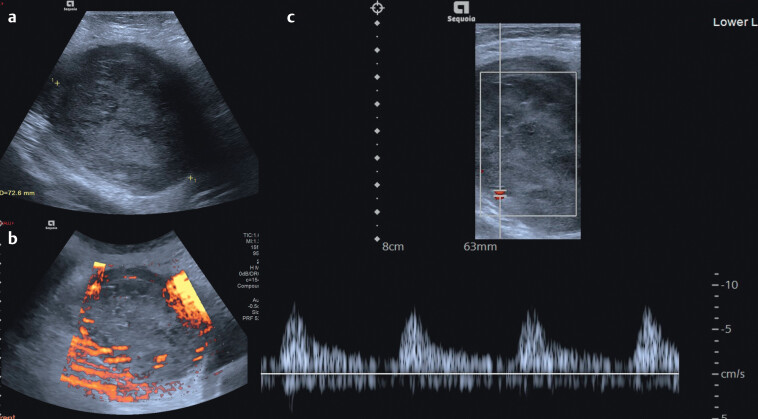
**(a and b)** Transverse B-mode extended field of view imaging demonstrates a 7.2 cm well-circumscribed left groin lesion with heterogenous echotexture and marked peripheral and intra-lesion vascularity. ( **c** ) Transverse pulse-wave Doppler imaging targeted on an intra-lesional vessel demonstrates arterial flow. The Doppler gain was increased, and the scale reduced to maximise vascular signal from the central portions of the tumour hence producing an image with additional noise.

Subsequently, a focused ultrasound was performed using a curvilinear multi-frequency probe (1–7 MHz) and a linear probe (7–15 MHz), with a combination of M-mode and Doppler (colour, power and pulse-wave). This demonstrated a 7.2 cm, well-circumscribed, left groin lesion with heterogenous echotexture with increased peripheral and intra-lesion arterial doppler vascularity ( Fig. 2 ). The ultrasound images therefore were concerning for a primary soft tissue tumour, over the alternative working diagnoses.

**Fig. 3:**
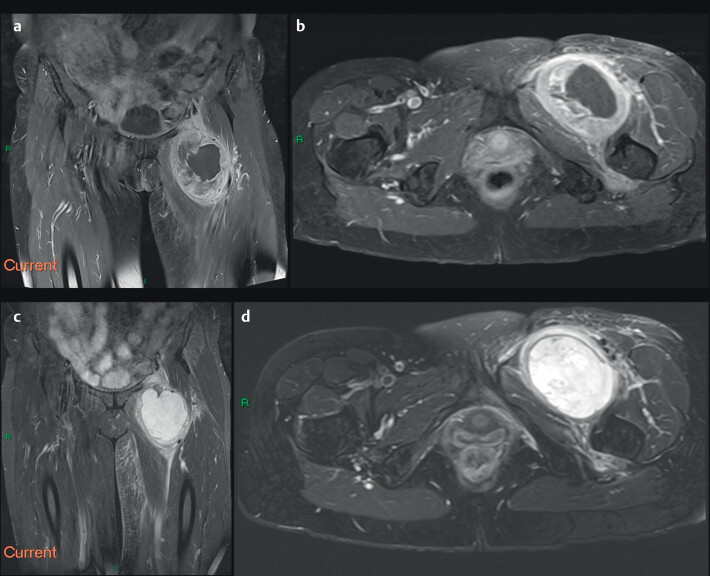
**(a and b)** MRI pelvis with post-gadolinium enhanced T1-weighted axial and coronal sequences. Post-contrast images reveal the marked circumferential enhancement of the nodular soft tissue components of the lesion. There is a large central region of low signal intensity consistent with intra-tumoural necrosis. ( **c and d** ) MRI pelvis with T2-weighted fat-suppressed coronal and axial sequences again shows a large, heterogenous intermuscular mass in the medial compartment of the left upper thigh with associated mass effect and perilesional oedema. MRI, magnetic resonance imaging; PD, proton density.

**Fig. 4:**
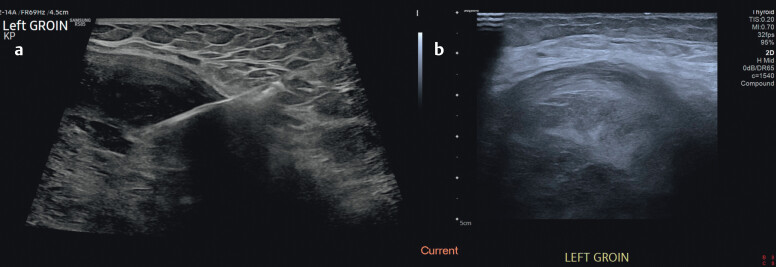
**(a and b)** Ultrasound-guided core biopsy of the described left medial groin mass using transverse B-mode imaging.

Following this, the patient urgently underwent a gadolinium-enhanced magnetic resonance imaging (MRI), which revealed a nodular, intermuscular mass with marked circumferential enhancement, a large region of central intra-tumoural necrosis, and intra-fascial extension ( Fig. 3 ). The patient was promptly referred to a tertiary centre for tissue sampling via ultrasound-guided biopsy ( Fig. 4 ), and the histopathology demonstrated features of an intramuscular myxoid sarcoma, favouring a diagnosis of MFS. They are currently undergoing definitive management with radiotherapy and surgical excision.

## Discussion

The initial evaluation of a soft tissue mass in the context of raised inflammatory markers can be diagnostically challenging.

CT can be useful in identifying fat and calcification, as well as help delineate the vascular anatomy, but is often non-specific. Appearances of lesions such as MFS may be indeterminate, such as this case, where the described CT features were suggestive of an abscess.

Ultrasound proved a highly useful adjunct in the initial assessment of this soft tissue mass, allowing for the differentiation of solid, cystic and myxoid components as well as the assessment of vascularity and compressibility. This underscores the cruciality of a comprehensive sonographic assessment, combined with a focused history and examination, for considering suspicious features of malignant soft tissue lesions such as MFS. Disorganised vascular patterns with abnormal morphology and flow characteristics are well-recognised markers of malignancy; however, US alone may underestimate deep extension or infiltrative margins which can be seen in MFS.

MRI remains the gold standard for local staging and characterisation of soft tissue sarcomas. MRI is highly specific for MFS and correlates with microscopic infiltrative disease extending beyond what may be appreciated intra-operatively or on gross examination. In particular, the mass also demonstrated tapering, enhancing extension along the fascia in keeping with the classic ‘tail sign’ (Lefkowitz, R.A. et al. in *Skeletal Radiol* , 2013). The tail sign is not exclusive to MFS and can occur in other benign myxoid processes such as nodular fasciitis and reactive myxoid proliferation as well as other malignant myxoid lesions such as myxoid liposarcoma or undifferentiated pleomorphic sarcoma. The patient’s prior clinical history of radiotherapy treatment for cervical cancer was also pertinent in considering radiation-induced sarcoma.

Histopathological analysis is essential for a definitive diagnosis. In this case, histopathology demonstrated a cellular tumour containing atypical stromal cells in a myxocollagenous matrix. The further adjacent tumour cores cellular contained dispersed, tumour mesenchymal cells in a richly myxoid matrix displaying highly atypical, pleomorphic nuclei. Supplementary immunochemistry revealed focally positive CD34 and again favoured FNCLCC grade 2 myxofibrosarcoma.

Management of MFS is guided by multidisciplinary, specialist care. Wide-local surgical excision is the primary treatment for local lesions, with radiotherapy used in the neoadjuvant or post-adjuvant setting to reduce the recurrence risk. Systemic therapies such as chemotherapy or immunotherapy may also be considered in certain cases. Early recognition of suspicious imaging features and prompt referral to specialist centres are important in ensuring timely staging and management.

MFS may be misdiagnosed due to atypical symptoms, an unusual location, or atypical imaging features. Recognising the ultrasound imaging features of malignant sarcomas is essential for detection, early diagnosis, and treatment. This case has shown the essential role of multi-imaging modalities in the characterisation of complex soft tissue lesions such as MFS.

